# Structural White Matter Correlates of the Crowding Effect: Insights From a Tractography Study of the Arcuate Fasciculus Post‐Hemispherotomy

**DOI:** 10.1002/hbm.70258

**Published:** 2025-06-19

**Authors:** Justus Bisten, Johannes Grün, Christian Hoppe, Tobias Bauer, Nina R. Held, Renata Rose, Anita Althausen, Juri‐Alexander Witt, Valeri Borger, Matthias Schneider, Hartmut Vatter, Christoph Helmstaedter, Alexander Radbruch, Rainer Surges, Thomas Schultz, Theodor Rüber

**Affiliations:** ^1^ Department of Neuroradiology University Hospital Bonn Bonn Germany; ^2^ Department of Epileptology University Hospital Bonn Bonn Germany; ^3^ Institute for Computer Science, University of Bonn Bonn Germany; ^4^ Bonn‐Aachen International Center for Information Technology (b‐It) Bonn Germany; ^5^ German Center for Neurodegenerative Diseases (DZNE) Bonn Germany; ^6^ Institute of Biochemistry and Molecular Biology, University Hospital Bonn Bonn Germany; ^7^ Center for Medical Data Usability and Translation (ZMDT) Bonn Germany

## Abstract

The neuropsychological crowding effect denotes the reallocation of cognitive functions within the contralesional hemisphere following unilateral brain damage, prioritizing language at the expense of nonverbal abilities. This study investigates structural white matter correlates of crowding in the arcuate fasciculus (AF), a key language tract, using hemispherotomy as a unique setting to explore structural reorganization supporting language preservation. We explore two main hypotheses. First, the contralesional right AF undergoes white matter reorganization correlated with preserved language function at the expense of nonverbal abilities following left‐hemispheric damage. Second, this reorganization varies with epilepsy etiology, influencing different stages of developmental language lateralization. This retrospective study included individuals post‐hemispherotomy and healthy controls. Inclusion criteria were; (1) being a native German speaker, (2) having no MRI contraindication, (3) the ability to undergo approximately 2 h of MRI scans, and (4) the ability to participate in neuropsychological assessments over two consecutive days. Neuroimaging included T1‐, T2‐, and diffusion‐weighted imaging, alongside postoperative neuropsychological assessments, where it was taken as evidence for crowding if verbal IQ exceeded performance IQ by at least 10 points. The AF was reconstructed using advanced tractography, and CoBundleMAP was used to compare morphologically corresponding AF subsections. Statistical significance was set at p<0.05, with correction for multiple comparisons applied across contiguous tract sections using Threshold‐Free Cluster Enhancement. The final cohort comprised 22 individuals post‐hemispherotomy (median age: 20.4 years, range: 12.3−43.9; 55% female; 55% with left‐sided surgeries) and 20 healthy controls (median age: 23.8 years, range: 15.5−54.0; 55% female). Crowding was associated with significantly higher fractional anisotropy (FA) in the AF (p=0.015, Cohen's d=1.69), but only observed in individuals with left‐sided hemispherotomy, localized to a subsection between Geschwind's territory and Wernicke's area ($p_{\text{corrected}}=0.02$). This region also displayed significantly higher normalized FA in AF of individuals with congenital etiology and crowding compared to acquired etiology and no crowding ($p_{\text{corrected}}=0.0189$). This study identifies previously unreported neural correlates of crowding in right contralesional AF of individuals post‐hemispherotomy and highlights specific AF subsections involved in preserving language functions at the cost of nonverbal abilities. The findings suggest a link between crowding and epilepsy etiology, particularly in the region spanning Geschwind's territory and Wernicke's area.

## Graphical abstract

This study examines white matter integrity in individuals post‐hemispherotomy and healthy controls using diffusion MRI tractography and CoBundleMAP, which aligns tract segments across hemispheres and subjects within a shared manifold. Our findings reveal fractional anisotropy differences in the contralesional right arcuate fasciculus associated with the crowding effect and epilepsy etiology.

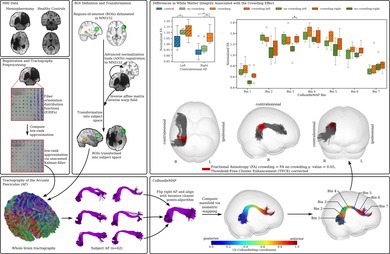

## 1 Introduction

Numerous studies have demonstrated that cerebral lesions, such as those commonly seen in refractory epilepsy, can prompt remarkable neuroplastic responses both degenerative and adaptive in nature (Bauer et al. 2024; Gaubatz et al. 2020; Held et al. 2023). It is these responses, which appear to enable significant functional recovery even after substantial neural disruptions and facilitate the brain's remarkable ability to compensate for large lesions and pathological insults, sometimes even through means of functional redundancy (Prillwitz et al. 2021). However, previous studies of individuals with epilepsy suggest that some functions can only be retained at the cost of sacrificing others. Children with drug‐resistant epilepsy (DRE) frequently exhibit neurocognitive decline, particularly in language functions, which significantly impacts academic achievement, social integration, and may predispose individuals to long‐term occupational and psychological difficulties, ultimately reducing overall quality of life (Jeong et al. 2024; Kalilani et al. 2018; Wang et al. 2019). In this context, the retention of language functions has long been associated with a loss of nonverbal functionality (Jeong et al. 2024; Lidzba et al. 2006; Loring et al. 1999; Strauss et al. 1990). More specifically, studies on children with early left‐hemispheric lesions and DRE have reported deficits in visuo‐spatial tasks or lower performance IQ (PIQ) (Helmstaedter et al. 1994; Jeong et al. 2024; Muter et al. 1997; Staudt et al. 2002; Vargha‐Khadem et al. 1994). Neuropsychologically, this is attributed to the concept of crowding, whereby the preservation of left‐hemispheric language functions is achieved through compensatory recruitment of the right hemisphere, resulting in a trade‐off that compromises nonlinguistic functions typically mediated by the right hemisphere (Helmstaedter, Kurthen, Gleißner, et al. 1997; Lansdell 1969; Teuber 1974). In this scenario, multiple cognitive functions co‐opt neural resources of the remaining right hemisphere, leading to the “crowding out” of those deemed less important, following left‐hemispheric damage (Helmstaedter et al. 1994; Lidzba et al. 2006; Teuber 1974). Crowding could serve as a crucial mechanism of language reorganization and preservation in children with DRE and left‐hemispheric lesions (Jeong et al. 2024). Thus far, no neuroimaging studies have delineated specific patterns of white matter microstructure, such as diffusion alterations indicative of localized changes in tract integrity, which are associated with the crowding effect.

Language processing relies on a distributed network of white matter pathways, including the inferior fronto‐occipital fasciculus (IFOF), the uncinate fasciculus (UF) which have been shown to be involved in semantic processing (Catani et al. 2013; Martino et al. 2010),, the superior longitudinal fasciculus III (SLF‐III), which has been attributed a central role in acoustic phonological encoding including word perception (Giampiccolo and Duffau 2022), and the arcuate fasciculus (AF). The AF is one of the primary white matter structures involved in language function. It is a prominent white matter tract that arches between Broca's and Wernicke's areas, as well as between their contralateral homologues (Broca et al. 1861; Catani et al. 2005; Wernicke 1874). The left‐hemispheric AF has long been suggested to play a central role in language processing (Dick and Tremblay 2012; Geschwind 1970; Giampiccolo and Duffau 2022; Konorski et al. 1961), particularly in speech production and perception (Dick and Tremblay 2012; Warren et al. 2005). Damage to this tract has repeatedly been associated with language impairments, underscoring its importance in the neural language network (Ivanova et al. 2021; Jenkinson et al. 2012). In cases where lesions are extensive and associated with drug‐resistant epilepsy, surgical interventions such as hemispherotomy are sometimes necessary (Delalande 1992). Hemispherotomy is considered a last‐resort intervention for drug‐resistant catastrophic epilepsy (Delalande et al. 2007). It is predominantly proposed in children with catastrophic epilepsy associated with a congenital or acquired hemispheric cerebral pathology (Delalande et al. 2007). With its complete disconnection of the ipsilesional hemisphere, hemispherotomy brings forth a unique setting for studying cerebral plasticity in which integrity and cognitive functions can solely be attributed to the contralesional hemisphere (Pinabiaux et al. 2022). Studies have reported commendable cognitive outcomes and emphasized the brain's remarkable capacity to recover language networks following left hemispherotomy, accompanied by a reorganization of language networks toward the right hemisphere (Pinabiaux et al. 2022). CoBundleMAP is a tractography analysis method that employs manifold learning to achieve consistent parameterization of white matter fiber bundles, offering both one‐ and two‐dimensional options depending on the bundle's structure (Khatami et al. 2017; Khatami et al. 2019). By focusing on core sections of the tract, CoBundleMAP is able to facilitate a localized analysis of structural integrity metrics such as fractional anisotropy (FA) along the white matter tract. For this study, we leverage this ability to establish precise anatomical correspondences between the left and right hemispheres across all subjects, enabling direct comparisons between the AFs of individuals who underwent left‐ and right‐hemispherotomy, as well as those of healthy controls. Numerous studies have demonstrated that alterations in white matter integrity can be spatially restricted along specific segments of fiber tracts, rendering whole‐tract averaging insufficiently sensitive to such localized changes (Colby et al. 2012; Joo et al. 2025; Qiu et al. 2025; Yeatman et al. 2012). This underscores the importance of anatomically precise, along‐tract analyses such as those enabled by CoBundleMAP, which allow for the detection of focal microstructural abnormalities potentially linked to neuroplastic reorganization mechanisms like crowding.

This study investigates two main hypotheses. First, we hypothesize that in individuals with epilepsy who undergo left‐sided hemispherotomy, crowding facilitates compensatory white matter reorganization concerning the homologous brain areas of Broca's and Wernicke's areas in the right contralesional hemisphere. Correspondingly, we exclusively expect the right contralesional AF arching between both areas to exhibit localized increases in imaging markers for white matter integrity, such as FA. Furthermore, we anticipate these increases to be correlated with the extent of language function preserved at the expense of nonverbal cognitive function. Second, we propose that the extent and localization of neuroplastic reorganization within the AF may vary based on etiology, as in healthy children, left‐hemispheric language dominance is typically established before age 5 or 6 (Jeong et al. 2024; Szaflarski et al. 2006). In this developmental stage, lateralization is driven through processes like synaptic pruning and increased myelination within the left‐hemisphere's temporal and frontal language regions (Chou et al. 2018; Danguecan and Smith 2019; Jeong et al. 2024). Consequently, we hypothesize that the congenital or acquired nature of the individuals' epilepsy may critically influence the brain's capacity for plasticity and crowding as language functions reorganize in response to left‐hemispheric damage.

In a related study, Jeong et al. investigated the crowding effect in children with left‐hemispheric drug‐resistant epilepsy, focusing on network‐level connectivity patterns in verbal and nonverbal modular networks (Jeong et al. 2024). Their study used a unique diffusion‐weighted connectome approach to identify local efficiency as a marker of inter‐hemispheric language reorganization across multiple pathways. They identified several eloquent areas as hubs for both verbal and nonverbal cognitive processing. One white matter pathway emanating from these hubs was the AF. Extending on their work, our study exclusively targets the AF as the primary language tract, using hemispherotomy as a unique model to study long‐term compensatory reorganization, which can be solely attributed to the contralesional hemisphere. By focusing on sectional analyses of the AF (Figure 6), our approach provides precise localization of structural changes, offering insights distinct from broader network‐level assessments.

## 2 Results

### 2.1 Participants

The study initially enrolled 20 healthy controls and 34 individuals who underwent hemispherotomy between 1992 and 2012. Nine individuals post‐hemispherotomy were excluded due to their inability to complete the imaging protocol. Following further exclusions due to missing neuropsychological data for two post‐hemispherotomy individuals and unsuccessful AF reconstruction during tractography for another, the final cohort is comprised of 42 participants: 22 individuals post‐hemispherotomy and 20 healthy controls, matched for age and sex (Table 1). The patient and control cohorts do not significantly differ in age at the time of the scan (p=0.125, independent sample t‐test). The median age at the time of the scan is 20.4 years (range 12.3−43.9) for individuals post‐hemispherotomy and 23.8 years (range 15.5−54.0) for healthy controls. Within the post‐hemispherotomy group, the majority has a congenital etiology (77%, *n* = 17) and underwent left‐sided hemispherotomy (55%, *n* = 12). The distribution of individuals assigned male or female at birth is similar between groups (p=1.0, Fisher's exact test). The median age at seizure onset is 3.5 years, with a median epilepsy duration of 9.2 years before surgery. The median age at surgery is 12.15 years, and the median postsurgical interval at the time of scanning is 9.6 years. Long‐term seizure freedom (≥1 year post‐surgery) is reported in 91% of the patients (n=20), while seizure frequency prior to surgery is available for 18 individuals and categorized into four clinical severity groups. To assess cognitive outcomes, we stratify the post‐hemispherotomy group based on the presence of crowding. The crowding group shows higher VIQ (85.5 [69.0–110.0], mean [range]) and lower PIQ (64.5 [54.0–91.0]) compared to the no‐crowding group (VIQ: 71.5 [51.0–89.0], PIQ: 73.5 [45.0–100.0]). These values indicate that both subgroups perform below normative means.

**TABLE 1:** Participant demographics and IQ scores.

	Individuals post‐hemispherotomy	Healthy controls	*p*
Subjects, *n*	22	20	n.a.
Sex female, *n* (%)	12 (55)	11 (55)	1.0
Etiology congenital, *n* (%)	17 (77)	n.a.	n.a.
Age at scan; years, median (range)	20.4 (12.3–43.9)	23.8 (15.5–54.0)	0.125
Age at surgery; years, median (range)	12.15 (0.7–33.9)	n.a.	n.a.
Age at seizure onset; years, median (range)	3.5 (0.0–10.0)	n.a.	n.a.
Side of hemispherotomy left, *n* (%)	12 (55)	n.a.	n.a.
Duration of epilepsy; years, median (range)	9.2 (0.7–30.9)	n.a.	n.a.
Postsurgical interval; years, median (range)	9.6 (1.2–19.4)	n.a.	n.a.
Seizure freedom postsurgery, *n* (%)	20 (91)	n.a.	n.a.
Seizure frequency category, *n* (%)			
≥ 1 per day	11 (50)	n.a.	n.a.
< 1 per day but ≥ 1 per week	4 (18)	n.a.	n.a.
< 1 per week but ≥ 1 per month	3 (14)	n.a.	n.a.
not reported	4 (18)	n.a.	n.a.
VIQ; score, median (range)			
Crowding	85.5 (69.0–110.0)	n.a.	n.a.
No crowding	71.5 (51.0–89.0)	n.a.	n.a.
PIQ; score, median (range)			
Crowding	64.5 (54.0–91.0)	n.a.	n.a.
No crowding	73.5 (45.0–100.0)	n.a.	n.a.

*Note:* Summary of demographic, clinical, and cognitive data for individuals post‐hemispherotomy and healthy controls. An independent sample t‐test evaluates group differences in age at the time of the MRI scan, and Fisher's exact test assesses group differences in sex assigned at birth. Cognitive outcomes (VIQ, PIQ) are stratified by the presence of crowding in the post‐hemispherotomy group.

Abbreviation: N.a., not applicable.

### 2.2 Integrity of the AF in Epilepsy Patients Post‐Hemispherotomy

**FIGURE 1:**
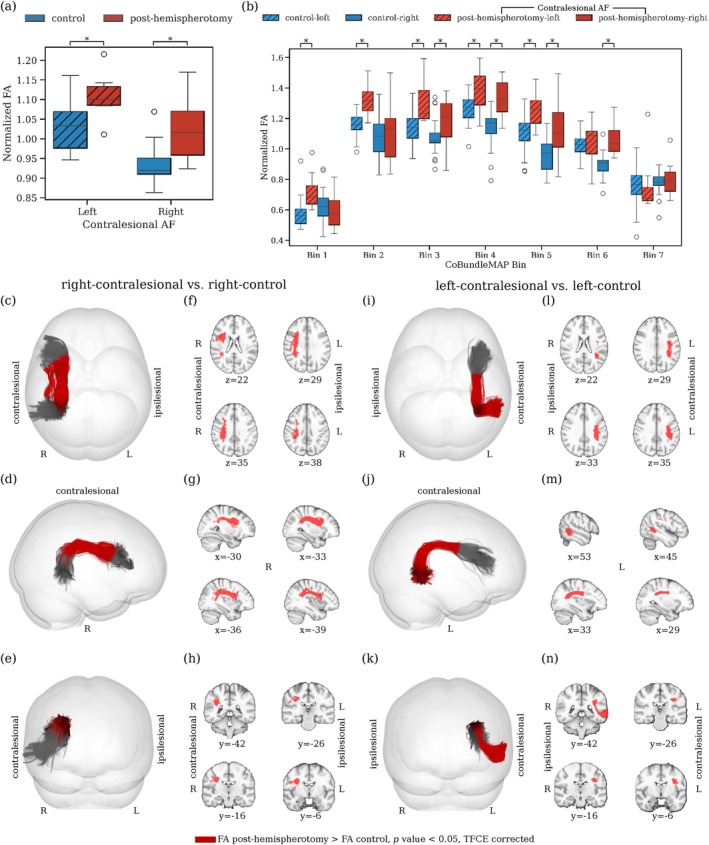
Normalized FA along the AF for individuals post‐hemispherotomy and controls. (a) Comparison of whole‐tract normalized FA between individuals post‐hemispherotomy and controls. (b) Comparison of normalized FA along the tract via CoBundleMAP. Bins with significant difference between individuals post‐hemispherotomy and controls are overlaid in red on the joint AF tractographies from the respective hemispheres ((c–e), right contralesional; (i–k), left‐contralesional). Corresponding slices (MNI152) are displayed for both contralesional hemispheres ((f–h), right; (l–n), left). Asterisks denote bins with statistically significant differences between groups ($p_{\text{corrected}}<0.05$) after TFCE correction for multiple comparisons.

Whole‐tract FA is consistently higher in individuals post‐hemispherotomy compared to controls for both hemispheres (left contralesional, p=0.0017, Cohen's d=1.48; right contralesional, p=0.0007, Cohen's d=1.34, Figure 1a). The sectional analysis of the AF reveals significant differences in normalized FA between individuals post‐hemispherotomy and controls. For the left hemisphere, normalized FA in the contralesional AF is significantly higher in individuals post‐hemispherotomy compared to controls across bins 1 to 5 (all $p_{\text{corrected}}\leq 0.008$). However, in the right hemisphere, normalized FA in the contralesional AF is significantly higher in individuals post‐hemispherotomy compared to controls across bins 3 to 6 (all $p_{\text{corrected}}\leq 0.007$, Figure 1b). These findings reveal a pattern of increased white matter integrity in the contralesional AF of individuals post‐hemispherotomy relative to controls, with the effect varying across tract sections and with respect to the contralesional side (Figure 1c–n). Further diffusion metrics such as mean diffusivity (MD), axial diffusivity (AD), and radial diffusivity (RD) did not show any significant differences between groups irrespective of the contralesional side (all $p_{\text{corrected}}>0.05$, TFCE‐corrected; data not shown).

### 2.3 Crowding‐Associated Differences in FA of the AF Post‐Hemispherotomy

**FIGURE 2:**
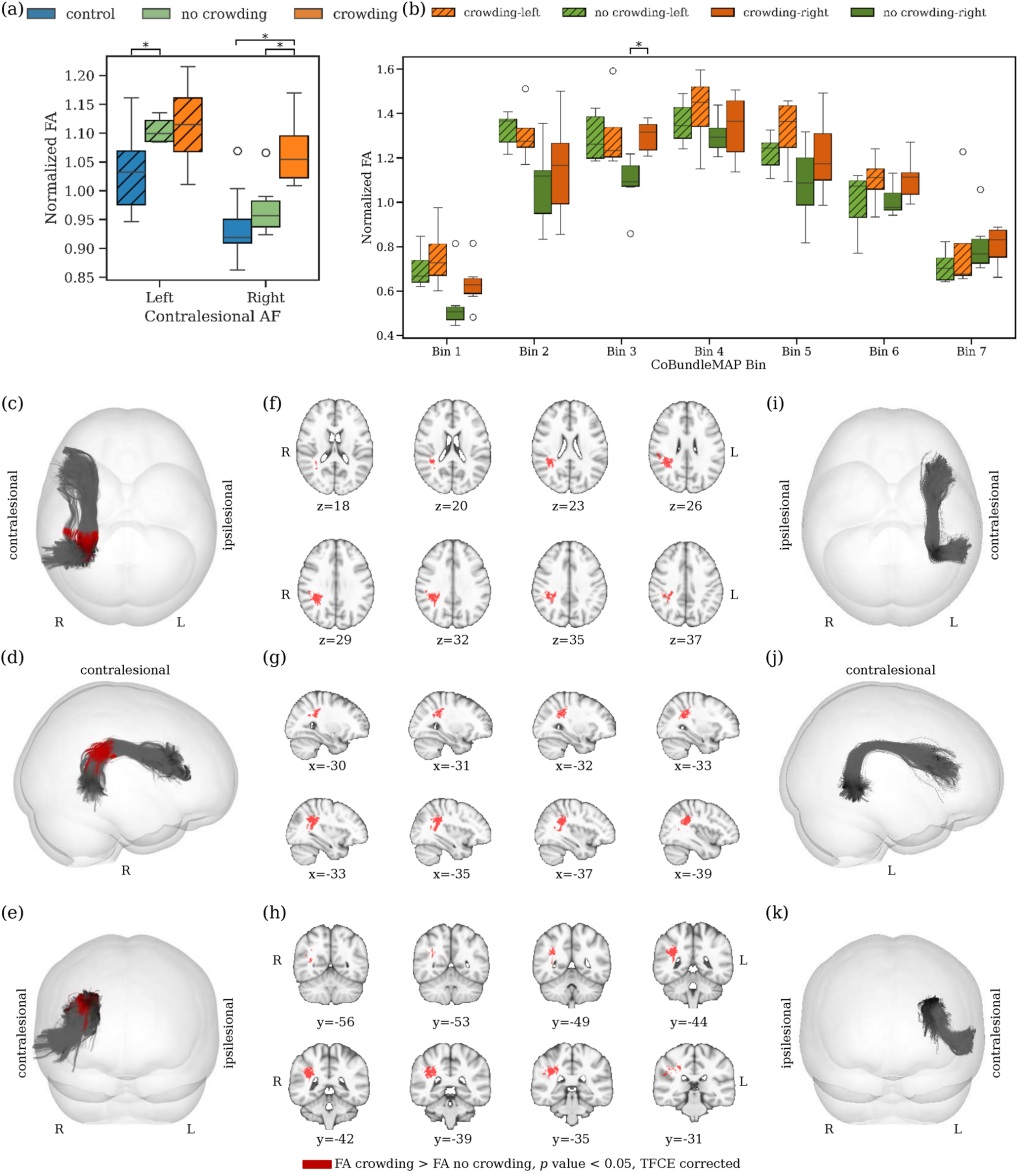
Crowding‐related differences in FA of the AF. (a) Whole‐tract normalized fractional anisotropy (FA) for the crowding, no‐crowding, and control subgroups. (b) Sectional analysis of normalized FA using CoBundleMAP for the crowding and no‐crowding subgroups. Bins with significant difference between individuals with crowding and individuals without crowding are overlaid in red on the joint AF tractographies from the respective hemispheres ((c‐e), right contralesional; (i–k), left‐contralesional). Corresponding slices (MNI152) are displayed for right contralesional hemispheres (f–h). Asterisks denote statistically significant differences ($p_{\text{corrected}}<0.05$) after TFCE correction.

**FIGURE 3:**
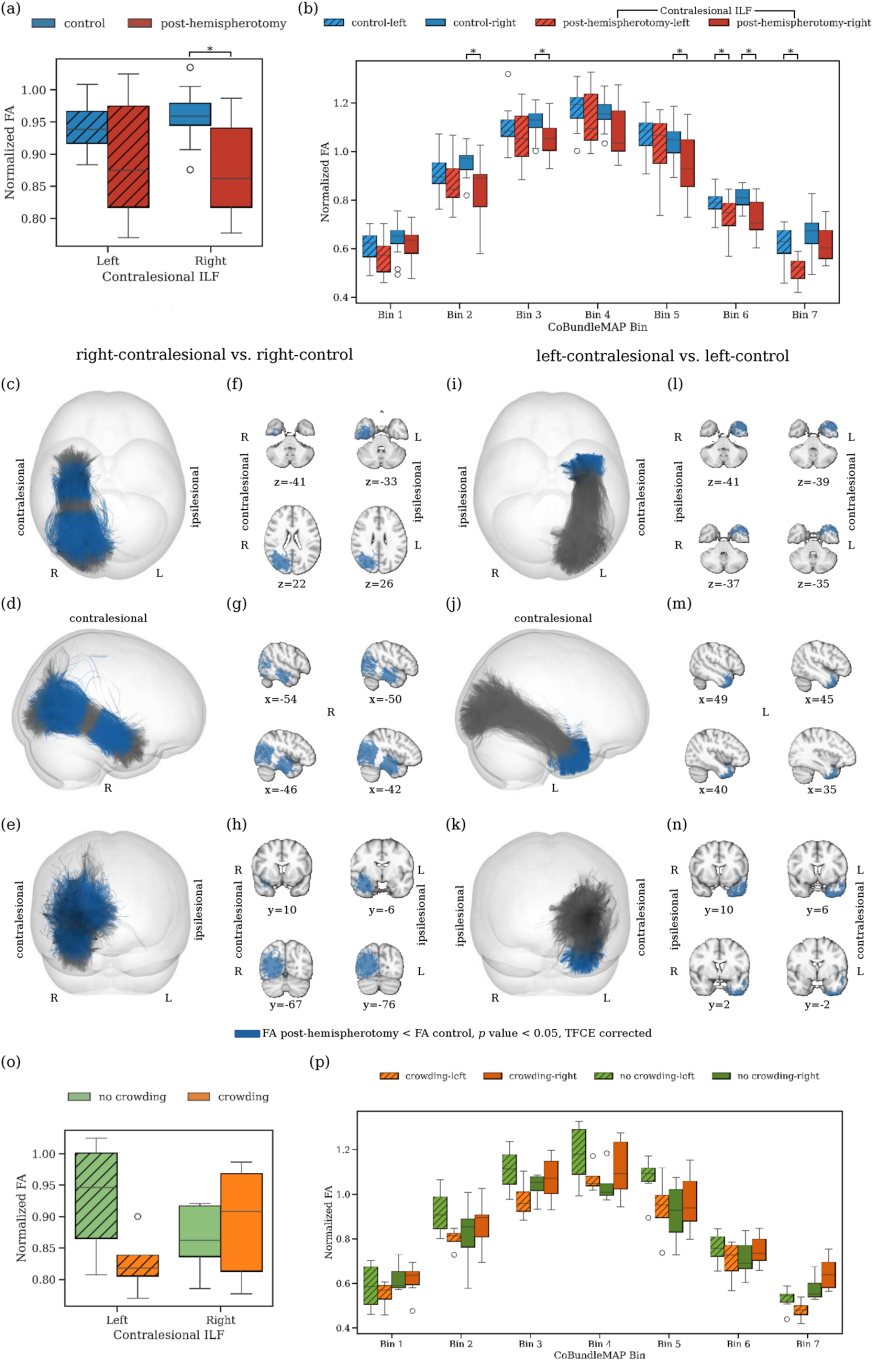
Specificity of crowding‐related differences with the ILF as a nonlanguage tract. (a) Whole‐tract normalized FA for the ILF post‐hemispherotomy, and control groups. (b) Section‐wise CoBundleMAP analysis of normalized FA of the ILF comparing healthy controls and individuals post‐hemispherotomy. Bins with significantly lower normalized FA are overlaid in blue along the ILF in both the right (c–h) and left (i–n) contralesional hemisphere. *p* values are corrected using TFCE. (o) Whole‐tract normalized FA and (p) section‐wise CoBundleMAP analysis of normalized FA of the ILF comparing individuals with crowding and no crowding.

The analysis of the AF, comparing crowding and no‐crowding subgroups within the post‐hemispherotomy cohort, reveals distinct patterns in normalized FA. For individuals with a contralesional left hemisphere, a significant whole‐tract difference in normalized FA between the no‐crowding, and control subgroup is observable (p=0.002, Cohen's d=1.53). There is no significant difference between the crowding and no‐crowding subgroup for left contralesional AF (p=0.76, Figure 2a). Contrary, for individuals with right contralesional hemispheres, the crowding subgroup exhibits significantly higher whole‐tract normalized FA compared to both the no‐crowding subgroup (p=0.015, Cohen's d=1.69) and controls (p=0.00006, Cohen's d=2.53). Using CoBundleMAP for the sectional analysis of the AF, a significant difference in FA between the crowding and no‐crowding subgroups can only be localized in individuals with right contralesional AF. In particular, significantly higher FA is observed for individuals with crowding in bin 3 of contralesional right AF (Figure 2b, $p_{\text{corrected}}=0.02$). This difference is localized in between Geschwind's territory and Wernicke's area (Figure 2c–h). For left contralesional AF, no other significant differences are found across bins for normalized FA (Figure 2i–k), and other diffusion metrics (AD, MD, RD, all $p_{\text{corrected}}$ > 0.05, not shown). Notably, no significant differences in local streamline curvature are apparent between crowding and no‐crowding subgroups for either left or right contralesional AF over all bins, indicating that the observed FA differences are unlikely to result from geometrical confounds. In contrast, complementary comparison of whole‐tract normalized FA in the ILF reveals a decrease in normalized FA in individuals post‐hemispherotomy compared to controls for right‐contralesional ILF (Figure 3a). In this context, CoBundleMAP reveals several bins with lower normalized FA in both right‐contralesional and left‐contralesional ILF (Figure 3b,c–n). However, there are no significant differences in whole‐tract normalized FA (left‐contralesional; p=0.17, right‐contralesional; p=0.66, Figure 3o) comparing individuals exhibiting crowding to those with no crowding. Moreover, no significant differences across any bin's diffusion metrics (FA, AD, MD, RD, all $p_{\text{corrected}}$ > 0.05, only FA shown, Figure 3p) can be observed comparing ILF of individuals exhibiting crowding and those who do not exhibit crowding. This indicates anatomical specificity of the findings with respect to crowding‐associated differences in normalized FA in right contralesional AF (Figure 2).

### 2.4 Predictive Modeling of Crowding Post‐Hemispherotomy

Logistic regression analysis across three models identified normalized FA as a consistent and significant predictor of crowding (p=0.038, p=0.034, p=0.035; Table 2). Among the tested specifications, the model including age at scan (Model 3) yielded the best fit, as indicated by the lowest BIC (26.02) and highest pseudo‐$R^{2}$ (0.55). In this model, both age at scan (p=0.028) and the affected hemisphere (right; p=0.045) emerged as significant covariates. In contrast, age at surgery (Model 1) and age at onset (Model 2) did not reach significance. All models demonstrated acceptable condition numbers (range: 3.31–3.34), which fall well below the threshold typically associated with multicollinearity concerns.

**TABLE 2:** Logistic regression models predicting crowding.

Model	Variable	Coefficient	Standard Error	*z*	*p*‐value	BIC	AIC	LLR *p*‐value	Pseudo *R* ^2^
Model 1	Constant	0.86	0.88	0.97	0.332	34.22	29.85	**0.0374**	0.279
	Age at surgery	0.90	0.57	1.58	0.114				
	Normalized FA	1.63	0.78	2.07	**0.038**				
	Affected hemisphere	−2.26	1.40	−1.62	0.105				
Model 2	Constant	1.21	1.04	1.16	0.248	33.70	29.34	**0.0296**	0.296
	Age at onset	1.05	0.61	1.71	0.088				
	Normalized FA	1.86	0.88	2.12	**0.034**				
	Affected hemisphere	−2.83	1.67	−1.70	0.090				
Model 3	Constant	2.37	1.44	1.64	0.101	26.02	21.65	**0.0008**	0.550
	Age at scan	3.60	1.63	2.20	**0.028**				
	Normalized FA	2.98	1.42	2.11	**0.035**				
	Affected hemisphere	−4.90	2.44	−2.01	**0.045**				

*Note:* Summary of three logistic regression models assessing the relationship between normalized FA and the presence of crowding, while controlling for clinical covariates. Each model includes normalized FA along with a different age‐related variable (age at surgery, age at onset, or age at scan) as well as affected hemisphere. Significant predictors are highlighted in bold. Model fit is summarized using AIC, BIC, pseudo‐$R^{2}$, and likelihood ratio test (LLR) *p*‐values.

### 2.5 Association Between Etiology and Crowding

The analysis of neuropsychological language and nonverbal test scores reveals significant differences in VIQ, and the delta (VIQ–PIQ) across etiology groups with respect to the laterality of the remaining AF in individuals post‐hemispherotomy (Figure 5o–q). For right contralesional hemispheres, individuals with congenital etiology exhibit significantly higher VIQ (p=0.0153, Figure 5o), a trend toward lower PIQ (p=0.19, not significant, Figure 5, (p)), and a significantly higher delta (p=0.0159, Figure 5q) compared to individuals with acquired etiology. No difference can be observed for individuals with a left contralesional hemisphere. Further, Barnard's exact test indicates a significant relationship between crowding and etiology (p=0.026), with no individuals exhibiting the combination of acquired etiology and crowding in this dataset (Figure 5n). Furthermore, CoBundleMAP analysis of normalized FA shows, that individuals with congenital etiology and crowding display significantly higher normalized FA in bin 3 compared to individuals with acquired etiology and no crowding ($p_{\text{corrected}}=0.0189$, Figure 5a). This pattern is consistent with prior observations of crowding‐related differences on the right remaining hemisphere, localized between Geschwind's territory and Wernicke's area (Figure 2).

## 3 Discussion

**FIGURE 4:**
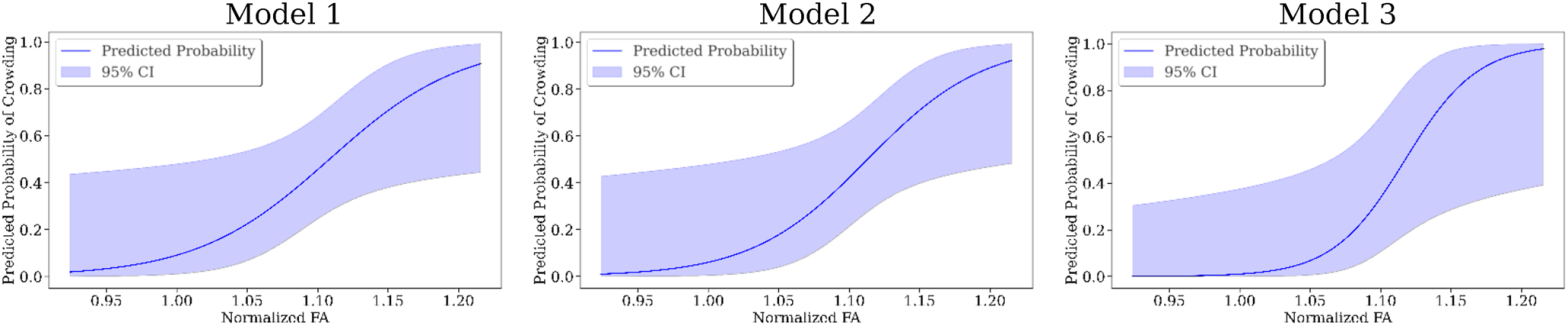
Predicted probability of crowding as a function of normalized FA for three logistic regression models. The solid blue line represents the predicted probability of crowding from the respective logistic regression model estimated from normalized FA. The shaded blue area indicates the 95% confidence interval.

**FIGURE 5:**
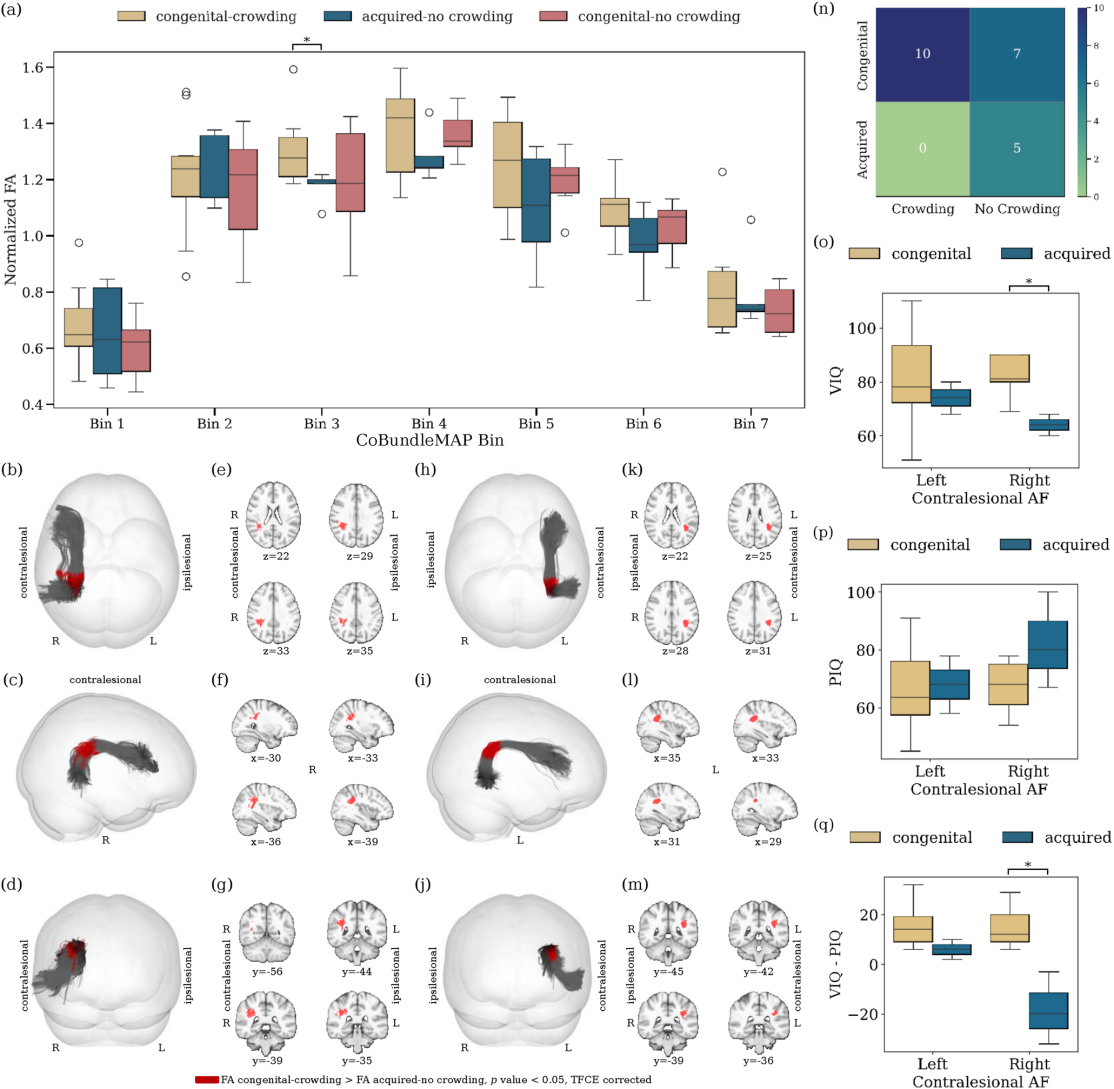
Verbal and nonverbal test performance in relation to etiology and normalized FA. (a) Sectional bin comparisons are shown for three groups: Congenital‐crowding, acquired‐no crowding, and congenital‐no crowding (all individuals post‐hemispherotomy, irrespective of surgery side). Bins showing significant differences between individuals with congenital etiology and crowding, and those with acquired etiology without crowding, are highlighted in red on the joint AF tractographies for the respective hemispheres ((b–d) right contralesional; (h–j) left contralesional). Corresponding MNI152 slices are provided for the right contralesional hemisphere (e–g) and the left contralesional hemisphere (k–m). (n) a 2 × 2 table showing the distribution of individuals by crowding and etiology. No cases of acquired‐crowding are present in this study group. Observed test scores for (o) VIQ, (p) PIQ, and (q) the delta (VIQ–PIQ), grouped by left and right contralesional hemisphere and further subgrouped by etiology (congenital and acquired) are shown. Statistically significant differences ($p_{\text{corrected}}<0.05$) after TFCE correction are marked with asterisks.

Our study provides distinctive insights into the structural reorganization within the AF in individuals post‐hemispherotomy. We observe higher normalized FA in the contralesional AF of hemispherotomy patients compared to controls, regardless of the side (Figure 1a); notably, with distinct sectional localization patterns on the AF (Figure 1b,c–h,k–n). In contralesional right AF, significantly higher normalized FA (Figure 1b,c–h) is observed in a section corresponding to the region where the AF resides in close proximity to and is partially overlapping with the third branch of the superior longitudinal fasciculus (SLF‐III) (Catani et al. 2005; Giampiccolo and Duffau 2022). It has been shown that speech arrest and articulation disorders can be induced subcortically via stimulation of the SLF‐III (Duffau 2015; Duffau et al. 2003; Giampiccolo and Duffau 2022; Maldonado et al. 2011), suggesting a central role in acoustic phonological encoding, including word perception (Giampiccolo and Duffau 2022). In left contralesional AF (Figure 1b,i–n) significantly higher normalized FA is apparent in the sections from the posterior inferior temporal gyrus (pITG) projections of the AF up to Wernicke's area and further into the connective region with the SLF‐III (Giampiccolo and Duffau 2022). These results reinforce preservation of the contralesional AF's structural integrity as a critical prerequisite for maintaining language following unilateral hemispheric insult and subsequent disconnection via hemispherotomy. Importantly, the crowding effect is hypothesized to occur solely in the right hemisphere following left‐hemispheric damage (Helmstaedter et al. 1994; Jeong et al. 2024; Lidzba et al. 2006; Teuber 1974). And while our findings highlight that the contralesional AF in both individuals with left‐ and right‐affected hemispheres may play a vital role in maintaining language function, we demonstrated higher normalized FA exclusively in the right contralesional AF of individuals with crowding compared to those without crowding (Figure 2a). This result supports our hypothesis that the right contralesional AF represents a potential neuroanatomical substrate for the crowding effect following left‐hemispheric damage. The observed differences were particularly localized to bin 3 of the sectional AF analysis (Figure 2c–h), corresponding to the segment of the AF between the right‐sided homologues of Wernicke's area and Geschwind's territory. This region is well established as a critical pathway for integrating higher order language functions, such as comprehension and production (Catani et al. 2005; Geschwind 1970). The localized increase in FA in this segment suggests that this portion of the AF may play a pivotal role in the reorganization required to support language functions in response to left‐hemispheric pathology. Furthermore, our findings are supported by several logistic regression models with normalized FA, the subject's age at scan, the subject's age at surgery, the subject's seizure onset and the laterality of the lesional hemisphere as the independent variables and crowding as the dependent variable (Table 2). The models persistently highlight normalized FA and thereby the structural integrity of the AF as a significant predictor of crowding. For increasing values of mean normalized FA of the AF, the models predict a higher probability for crowding in individuals post‐hemispherotomy (Figure 4). These findings provide evidence for a structural correlation between FA of the AF and crowding, suggesting the redistribution of neural resources in the right hemisphere, with the AF adapting to meet the demands of crowding by prioritizing language‐related processing over other cognitive functions. Our findings appear specific to the AF, as a corresponding analysis of the ILF, as a nonlanguage tract, reveals no significant differences in normalized FA between the crowding and no‐crowding subgroups (Figure 3g,p), albeit a slightly decreased normalized FA in individuals post‐hemispherotomy compared to controls (Figure 3a–n). Moreover, no significant differences are observed in local streamline curvature, MD, RD, or AD, between the crowding and no‐crowding groups underscoring the specificity of the effect to normalized FA. Our findings also shed light on the interaction between etiology and crowding‐associated structural reorganization. The significantly higher FA observed in congenital‐crowding cases relative to acquired no‐crowding cases (Figure 5, (a)), highlights the importance of developmental timing in shaping neuroplastic potential. This supports the notion that early brain insults, occurring before critical windows of plasticity during early childhood, enable more robust compensatory mechanisms compared to later, acquired insults (Helmstaedter et al. 1994, 1997). Notably, consistent with the sectional tract analysis of AF in individuals exhibiting crowding, significant differences can be observed in the region between Geschwind's territory and Wernicke's area (Figure 5b–g,j–m).

Clinically, these results emphasize the need to consider both crowding and etiology when assessing and predicting cognitive outcomes in hemispherotomy patients. They also suggest that neuroimaging markers for structural integrity of the AF, such as FA, could serve as a potential biomarker for language preservation facilitated by crowding, particularly in specific regions like Geschwind's territory and Wernicke's area (Figure 2c–h). This could inform pre‐ and postsurgical assessments and guide rehabilitation strategies aimed at optimizing outcomes for language and other cognitive domains. Furthermore, the findings underscore the importance of incorporating advanced imaging analysis methods, such as CoBundleMAP (Khatami et al. 2019), to capture localized white matter reorganization that might be obscured in whole‐tract assessments.

### 3.1 Limitations of the Study

The limited sample size constitutes a major constraint of this study, with direct implications for statistical power, particularly in subgroup analyses. The overall cohort size for individuals post‐hemispherotomy (*n* = 22) permitted core analyses. However, several subgroups, such as individuals with acquired etiology and no crowding, lacked substantial representation to support reliable inference. This limitation reduces the sensitivity to detect interaction effects or subtle group differences, and the generalizability of these results, in particular of the logistic regression models, should be considered with caution. Another key limitation is the absence of presurgical MRI and neuropsychological data in our study, which prohibits direct comparison between pre‐ and postoperative states. As a result, the observed differences in FA associated with the crowding effect may reflect a combination of preexisting pathology and postsurgical adaptation. Although all participants underwent postoperative assessments at least 1.2 years following surgery, the timing varied across individuals and was not standardized. The underlying variability may have influenced the extent to which longer term plastic changes were captured. Together, these factors limit the ability to differentiate whether behavioral and structural differences result from the hemispherotomy itself or reflect presurgical reorganization due to brain insult and epileptic seizures. Future prospective longitudinal studies are required to assess, whether reported differences in FA arise from preoperative pathology or postsurgical adaptation. In this context, a further limitation of this study is the inability to determine genuine preinsult language lateralization, particularly in cases of congenital etiology, where atypical language dominance may have existed prior to any crowding‐inducing pathological event. Additionally, nonlinear age‐related effects on neural plasticity and white matter development may have influenced the observed variability in structural and behavioral outcomes. Such effects are particularly relevant in pediatric populations, where brain maturation follows regionally heterogeneous and nonlinear trajectories (Alex et al. 2024). Future studies with larger samples should consider explicitly modeling age‐related plasticity using nonparametric approaches or age‐stratified analyses. Another potential limitation concerns the completeness of hemispheric disconnection, as residual interhemispheric connectivity or ongoing seizures could interfere with postsurgical language reorganization and confound behavioral outcomes. However, 91% of individuals in our cohort achieved long‐term seizure freedom (Table 1), indicating a high likelihood of functionally complete hemispheric disconnection and minimizing the risk of residual seizure activity or contralateral hemisphere influence on postoperative neuropsychological measures. Two individuals in our cohort experienced postoperative seizures, which, despite lacking evidence of incomplete disconnection or bilateral pathology, may have influenced cortical reorganization and represent a potential source of variability in structural outcomes. Furthermore, the definition of crowding in this study, VIQ exceeding PIQ by ≥ 10 points, reflects relative verbal preservation rather than normative performance, an approach likely appropriate for individuals post‐hemispherotomy who typically exhibit global cognitive impairment due to early‐onset pathology and surgical disconnection (Althausen et al. 2013; Bajer et al. 2020). While language abilities are often relatively spared in this population, they generally remain below typical developmental levels (Loring et al. 1999). In this study, both the crowding and no‐crowding groups scored below normative means, indicating that the VIQ–PIQ discrepancy reflects within‐subject cognitive asymmetry rather than intact cognitive functioning. Thus, the crowding definition in this study captures a relative cognitive profile within a globally low‐functioning cohort, which may be more appropriate than relying on normative benchmarks. Nonetheless, the lack of resolution with respect to normative performance may limit the generalizability of our findings to the broader population. Notably, the exact notions of SLF/AF connectivity and cortical termination are controversial and sometimes conflicting (Giampiccolo and Duffau 2022), highlighting the need for careful interpretation of our findings concerning the localization of FA differences, particularly in the context of their potential variability across methodologies and individual differences. Epilepsy is increasingly recognized as a network disease (Bernhardt et al. 2015; Galovic 2023; Kramer and Cash 2012), and language processing engages a distributed system of white matter pathways beyond the AF, including the IFOF and UF, both involved in semantic processing (Catani et al. 2013; Martino et al. 2010), as well as the SLF‐III, implicated in phonological encoding and word perception (Giampiccolo and Duffau 2022). In this study, isolating the AF allows precise mapping of localized structural adaptations and their association with behavioral outcomes. This complementing broader network‐level observations such as recent work by Jeong et al. (2024), who demonstrated the crowding effect at broader a network level. Future work should integrate both levels of analysis to delineate how tract‐specific reorganization interacts with system‐wide plasticity within the language network.

Despite these limitations, our findings provide the first evidence of structural correlates of the crowding effect in the contralesional right AF of individuals with left‐hemispheric pathological insult, offering a robust foundation for future research on its neuroplastic reorganization. By leveraging advanced imaging techniques and identifying localized structural changes, this study highlights regions that may warrant further investigation regarding their potential role in postsurgical language outcomes. Our findings emphasize the importance of considering the crowding effect, etiology, and developmental timing when evaluating postsurgical outcomes. Longitudinal‐ and presurgical imaging studies, as well as network‐level analyses, are needed to elucidate the temporal dynamics and broader connectivity implications of the underlying structural reorganization for cognitive function and its implications for cognitive and clinical management.

## 4 Methods and Materials

### 4.1 Experimental Model and Study Participant Details

The data for this study were selected retrospectively and involved individuals with epilepsy from the Department of Epileptology, University Hospital Bonn, who underwent hemispherotomy for medically refractory epilepsy, as well as healthy controls. Inclusion criteria were (1) being a native German speaker, (2) having no MRI contraindication, (3) the ability to undergo approximately 2 h of MRI scans, and (4) the ability to participate in neuropsychological testing over the course of 2 days. Data collection for this cohort occurred between 2011 and 2014. Individuals with epilepsy were classified into two etiology subgroups: congenital (porencephaly, hemimegalencephaly, and polymicrogyria) and acquired (Sturge–Weber syndrome, Rasmussen's encephalitis, ganglioglioma). This study was approved by the institutional review board of the University Hospital Bonn. All participants provided written and informed consent either personally or through a legal guardian.

### 4.2 MRI Acquisition

Images were acquired using a Siemens Healthineers Magnetom Trio 3 T MRI scanner equipped with an 8‐channel head coil between 2011 and 2014. The acquisition protocol was comprised of whole brain T1‐, T2‐, and diffusion‐weighted images. T1‐weighted images were acquired using an MP‐RAGE sequence comprised of 160 slices at a resolution of 1.0×1.0×1.0 mm^3^. The 3D‐T2‐weighted images were collected at a resolution of 1.0×1.0×1.0 mm^3^ (192 slices). Diffusion‐weighted imaging (DWI) was performed using single‐shot, dual‐echo, spin‐echo EPI sequences. The DWI acquisition sequence produced 72 axial slices at a resolution of 1.72×1.72×1.7 mm^3^. Diffusion‐weighting was applied along 60 directions with a b‐value of 1000 s/mm^2^, along with six baseline volumes (b=0 s/mm^2^).

### 4.3 MRI Preprocessing

MRI data were brought into a structure conforming to the Brain Imaging Data Structure format (Gorgolewski et al. 2016). Further preprocessing was facilitated using the FSL toolbox version 6.0.7 (Jenkinson et al. 2012). DWI data were denoised and subjected to eddy correction. The mean b=0 s/mm^2^ image was extracted using *dwiextract*. Skullstripping of the native T1‐images was facilitated utilizing a combination of FSL's *robustfov* and *bet2*. The skullstripped native T1‐images were normalized to the DWI space using Advanced Normalization Tools (ANTs) (Klein et al. 2009). White matter, gray matter and cerebral spinal fluid were segmented using FMRIB's Automated Segmentation Tool (Zhang et al. 2001). We employed FSL's *dtifit* to compute 3D‐images containing the FA. Fiber orientation distribution functions (fODFs) were then computed using the methodology described by Ankele and colleagues (Ankele et al. 2017).

### 4.4 Tractography of the AF and ILF

**FIGURE 6:**
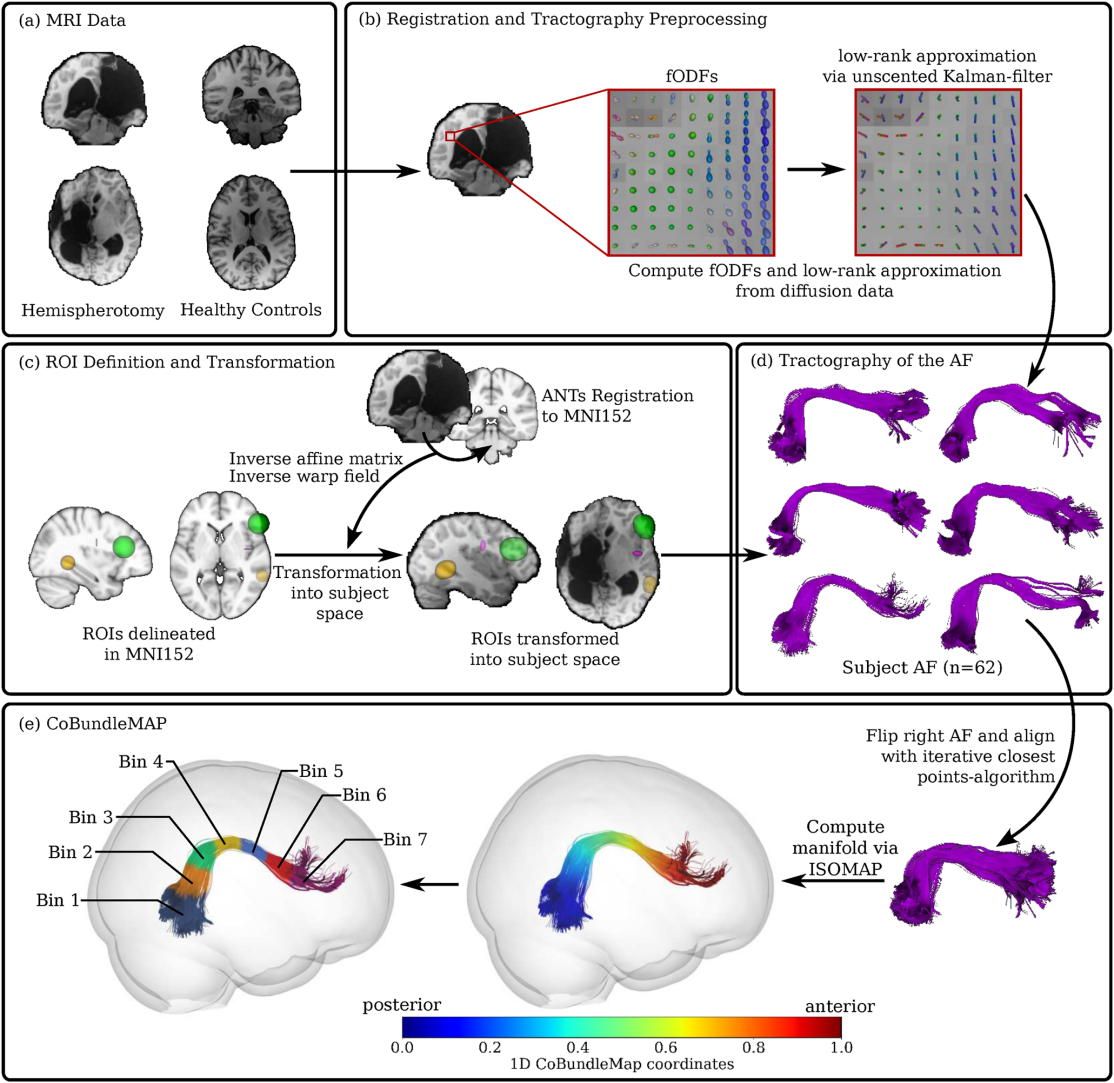
Methods Overview. Diffusion‐weighted MRI data of individuals with hemispherotomy and healthy controls (a) were used to compute fiber orientation distribution functions (fODFs) and low‐rank approximations (b) using an unscented Kalman filter. (c) Regions of interest (ROIs) for the AF were delineated in MNI152 space and transformed into subject space using ANTs‐based inverse affine matrices and warp fields. (d) Following whole‐brain tractography, the AF was extracted for each subject's intact hemispheres (*n* = 62). (e) Following alignment of right‐ and left‐sided AF via an iterative closest points algorithm and the computation of a joint manifold via isometric mapping (Tenenbaum et al. 2000), CoBundleMAP was used to segment the AFs of all subjects into 7 morphologically corresponding bins along their posterior–anterior trajectories.

For the tractography of the subjects' AF, we employed a state‐of‐the‐art algorithm that estimates a low‐rank tensor approximation of fODFs with an unscented Kalman filter, leading to a spatial regularization (Grün et al. 2023; Schultz and Seidel 2008). The code is readily available under the *bonndit* package (https://github.com/MedVisBonn/bonndit). Seeding was conducted in 3 anatomical regions‐of‐interest (ROIs) as defined by Barbeau and colleagues (Barbeau et al. 2020). To minimize annotation bias, ROIs were initially delineated in MNI152 space and subsequently transformed into each subject's native space using both affine and nonlinear transformations. The warp fields and affine matrices required for these transformations were derived during the normalization of the subject's native images to the MNI152 template (Figure 6c). The delineation of the AF was performed by filtering for streamlines that terminated within both the 30 mm diameter (Figure 6c, green ROI) and 20 mm diameter (Figure 6c, yellow ROI) endpoint spheres and traversed the “waypoint” ROI (Figure 6c, magenta ROI), which targeted the AF streamlines in the fronto‐parietal white matter stem. MNI coordinates for these ROIs were as follows: the temporal sphere (Figure 6c, yellow ROI) was centered at MNI coordinates *x* = −60, *y* = −45, *z* = 0 in the left hemisphere and x = 60, y = −43, z = −1 in the right hemisphere, with a diameter of 20 mm. The frontal sphere (Figure 6c, green ROI) was centered at MNI coordinates *x* = −53, *y* = 27, *z* = 20 in the left hemisphere and *x* = 49, *y* = 27, *z* = 20 in the right hemisphere, with a diameter of 30 mm. The fronto‐parietal white matter stem ROI (Figure 6c, magenta ROI) was defined at the level of the central sulcus. The ILF was dissected using three anatomically defined ROIs based on descriptions by Forkel et al. one waypoint ROI in the occipital lobe, one in the temporal lobe, and one exclusion ROI in the external capsule to isolate the ventral pathway from adjacent tracts (Forkel et al. 2025). Thereby, streamlines connecting the occipital and temporal ROIs while avoiding the external capsule were retained. ROIs were defined in MNI152 space and transformed to each subject's native space using affine and nonlinear warps.

### 4.5 Sectional Tract Analysis via CoBundleMAP

For a sectional comparison of the AF of both the left and right hemisphere across all subjects, we utilized CoBundleMAP (Figure 6e) as described by Khatami et al. (Khatami et al. 2017; Khatami et al. 2019) Outlier streamlines were identified and removed using a one‐class support vector machine, with a threshold of ν=0.1, classifying 10% of the streamlines as outliers. For each tract, a joint parameterization was computed using the isometric feature mapping (ISOMAP) manifold learning algorithm (Tenenbaum et al. 2000), aligning individual subject streamlines to a latent low‐dimensional bundlecore. This approach is robust to moderate anatomical distortions and pathological deviations in the targeted bundle. In particular, by aligning representative streamlines across hemispheres using Iterative Closest Point (ICP) registration and learning a joint ISOMAP manifold from the union of transformed tracts, CoBundleMAP captures the shared geometrical structure despite individual variability. Structural abnormalities, such as bundle thinning, displacement, or partial truncation due to lesion or resection, result in incomplete but topologically consistent projections into the manifold. Because CoBundleMAP restricts analysis to the well‐supported bundle core and uses nonparametric interpolation from aligned representatives, it mitigates the influence of sparse or anomalous tract segments, thereby ensuring consistent bin‐wise comparisons across subjects (Khatami et al. 2019). Notably, we only analyzed the AF in the contralesional hemispheres post‐hemispherotomy. Thus, we did not expect any majorly deformed tracts compared to healthy individuals. A 1D parameterization of the AF was generated by normalizing the ISOMAP coordinates to [0,1] along the bundle's length. We employed the ICP registration (Besl and McKay 1992), to align representative streamlines from left and right hemispheres before joint parameterization. We focused on core regions of the bundles, excluding under‐represented sections based on vertex density thresholds or insufficient subject coverage. Between‐group comparisons within bins were conducted using nonparametric Mann–Whitney U tests. To correct for multiple comparisons over contiguous CoBundleMAP bins, we applied permutation testing combined with Threshold‐Free Cluster Enhancement (TFCE) (Smith and Nichols 2009). TFCE scores were computed for an array of z scores z corresponding to the bins. In particular, the TFCE scores were calculated by iterating over a range of threshold heights iΔh and identifying clusters that exceed iΔh,

(1) $$\text{TFCE}\left(z\right)=\sum_{i=0}^{\left\lfloor \frac{\max\left(z\right)}{\Delta h}\right\rfloor }\left(e{\left(i\Delta h\right)}^{E}\cdot {\left(i\Delta h\right)}^{H}\right)\cdot \Delta h,$$

where *z* is the array of *z* scores across all bins, $e\left(i\Delta h\right)$ represents the cluster extent after thresholding the U test's *z* scores at threshold height iΔh. The extent weighting *E* emphasizes broader clusters, while the height weighting *H* prioritizes regions with higher *z* scores. The step size Δh controls the granularity of *z* score thresholds, with smaller values allowing for a more granular cluster detection. We used the default parameters for the TFCE, E=0.5 and H=2, with Δh set to 0.01. From the set of all possible group‐label permutations S, we sampled N permutations $\sigma_{i},i\in \left\{1,\ldots ,N\right\}$. For each permutation $\sigma_{i}$ we computed an array of z scores $z^{\sigma_{i}}$. We then calculated the TFCE across all bins $\text{TFCE}\left(z^{\sigma_{i}}\right)$. To ensure that computed *p*‐values are corrected for family‐wise errors, the maximum TFCE scores across all bins for each permutation were used to construct the null distribution

(2) $$D_{0}=\left\{\max\left(\text{TFCE}\left(z^{\sigma_{i}}\right)\right)|i=1,2,\ldots ,N\right\}.$$

Finally, *p*‐values for each bin $j\in \left\{1,\ldots ,M\right\}$ were computed as

(3) $$p_{j}=\frac{1}{N}\sum_{i=1}^{N}1\left(\max\left(\text{TFCE}\left(z^{\sigma_{i}}\right)\right)\geq \mathrm{TFC}E_{j}\left(z^{\mathrm{obs}}\right)\right)$$

where M is the total number of bins, 1 is the indicator function, returning 1 if the condition inside is true and 0 otherwise, $\text{TFCE}\left(z^{\sigma_{i}}\right)$ are the TFCE scores of the given permutation and ${\text{TFCE}}_{j}\left(z_{\mathrm{obs}}\right)$ are the observed TFCE scores of the participants.

### 4.6 Neuropsychological Assessment and Crowding Model

The neuropsychological assessment included a range of tests: the Boston Naming test (Kaplan et al. 2001), the Token test (De Renzi and Vignolo 1962; Hartje et al. 1973), Verbal Fluency tasks (both semantic and phonemic) (Benton 1968), the Mehrfachwahl–Wortschatz test (MWT‐B, a German vocabulary test which requires identifying true words among pseudowords) (Lehrl 1977), and a speech comprehension subtest from the AAT (Huber et al. 1983). Additional measures included the vocabulary test (WT, a subtest of HAWIE‐R and HAWIK‐III) and IQ scores derived from the Wechsler tests, namely verbal IQ (VIQ) and PIQ (Tewes 1994; Tewes et al. 2000). The former summarizes the performance in the language‐related subtests while the latter summarizes the performance in nonlinguistic tests from the batteries. Notably, one individual's IQ was measured using only the short version of the full‐scale test (Schwarzkopf‐Streit 2000). In this study, we focus on VIQ and PIQ, defining crowding as a cognitive profile in which VIQ exceeds PIQ by at least 10 points. This operationalization reflects the preservation of typically left‐hemispheric language skills at the expense of right‐hemispheric nonlinguistic abilities, often observed after left‐hemispheric brain damage.

### 4.7 Quantification and Statistical Analysis

Previous studies have demonstrated that FA can vary based on age and educational background (Grieve et al. 2007; Madden et al. 2009). To address these potential confounding factors, we applied tract‐based spatial statistics (TBSS) to create a common mean FA skeleton that represents the core of major white matter tracts across all subjects (Smith et al. 2006). Individual FA values for each subject were then aligned and projected onto this skeleton, resulting in individual tract skeletons. FA values for the subject's AF were then normalized by dividing each subject's mean FA of the AF by the mean FA of the subject's individual tract skeleton within the corresponding hemisphere. For subjects who had undergone hemispherotomy, the ipsilesional hemisphere was excluded from analysis. In addition to FA, we conducted analogous analyses for MD, RD, AD, following identical normalization and statistical procedures. To evaluate whether geometrical changes of the AF may have influenced our analysis. For this purpose, each streamline was represented as a discrete sequence of 3D points ${\left\{x_{i}\in R^{3}\right\}}_{i=0}^{V-1}$, uniformly resampled to 50 vertices. Despite using endpoint ROIs, streamlines in our study may vary slightly in physical length. Thus, we consider the arc length spacing Δs individually for each streamline. Curvature $\kappa_{i}$ at each internal point i was then approximated using the magnitude of the second central difference with respect to arc length.

$$\kappa_{i}\approx \left\|\frac{x_{i+1}-2x_{i}+x_{i-1}}{{\left(\Delta s\right)}^{2}}\right\|$$

This approximation estimates the second derivative of position with respect to arc length. Curvature was undefined at the endpoints i=0 and i=V−1, and set to zero. To assess the interaction between etiology and crowding, we used Barnard's exact test (Barnard 1945). We employed logistic regression to evaluate predictors of crowding. Model selection was guided by the Bayesian Information Criterion (BIC) and assessment of the condition number of the design matrix, evaluating goodness of fit and verifying the absence of multicollinearity, respectively. The model included normalized FA, the subjects’ age at scan, the subjects’ age at surgery, and the laterality of the affected hemisphere (right vs. left) as explanatory variables. For the comparison of the IQ scores we used nonparametric Mann–Whitney U test. All statistical analyses were facilitated using *statsmodels*, *scipy*, and *seaborn*. An effect is regarded statistically significant if p<0.05. Correction via TFCE was applied where necessary. For the visualization of tractography results, the significance in CoBundleMAP bins and MRI image slices, we used *fury* (Garyfallidis et al. 2021).

## Author Contributions

Conceptualization, J.B., J.G., C.Ho., T.R., T.S.; methodology, J.B., J.G., C.Ho., T.S.; investigation, J.B.; formal analysis, J.B., J.G., T.S.; visualization, J.B.; software, J.B., J.G.; resources, V.B., M.S., H.V., C.Ho., A.A., R.R., J.‐A.W., C.He, T.R., T.S.; writing (original draft), J.B.; data curation, J.B., T.B., R.R., N.R.H; review and editing, all authors; project administration, T.S., T.R., A.R., R.S., C.He; supervision, T.S., T.R.; funding acquisition, T.S., T.R., A.R., R.S.

## Conflicts of Interest

J.A.W. reports personal fees from Eisai GmbH, UCB Pharma GmbH, and Jazz Pharmaceuticals Germany GmbH, outside the submitted work. R.S. has received personal fees as a speaker or for serving on advisory boards from Angelini, Bial, Desitin, Eisai, Jazz Pharmaceuticals Germany GmbH, Janssen‐Cilag GmbH, LivaNova, LivAssured B.V., Novartis, Precisis GmbH, Rapport Therapeutics, Tabuk Pharmaceuticals, UCB Pharma, UNEEG, and Zogenix. He is an editorial board member of Epilepsy and Behavior and associate editor of Epilepsia Open. These activities were not related to the content of this manuscript. A.R. serves on the scientific advisory boards for GE Healthcare, Bracco, Bayer, Guerbet, and AbbVie; has received speaker honoraria from Bayer, Guerbet, Siemens, and Medscape; and is a consultant for, and has received institutional study support from, Guerbet and Bayer. These activities were not related to the content of this manuscript. The remaining authors declare no competing interests.

## Data Availability

The participants' MRI data are not publicly available due to privacy or ethical restrictions and will be shared by the lead contact upon request. All original code has been deposited at Github under the URL https://github.com/jbisten/hemitracts and will be made publicly available as of the date of publication.
